# Skin sensory alteration and kneeling ability following cruciate retaining total knee arthroplasty are not affected by the incision position: A randomised controlled trial of simultaneous bilateral surgery

**DOI:** 10.1186/s40634-023-00695-9

**Published:** 2023-12-23

**Authors:** Sergio Barroso Rosa, Matthew Wilkinson, Peter McEwen, Levi Morse, Andrea Grant, Kenji Doma, Charles Haward, Matthew Rikard-Bell

**Affiliations:** 1 The Orthopaedic Research Institute of Queensland, Pimlico, QLD Australia; 2 Department of Clinical and Surgical Sciences, University of Las Palmas de Gran Canaria, Las Palmas, Canary Islands Spain; 3 Department of Orthopaedic Surgery, Royal Hobart Hospital and Calvary Care, University of Tasmania, Hobart, TAS Australia; 4 Department of Orthopaedic Surgery, Townsville University Hospital, James Cook University, Townsville, QLD Australia; 5 Department of Sports and Exercise Science, James Cook University, Townsville, QLD Australia; 6 Department of Orthopaedic Surgery, Box Hill Hospital, Eastern Health, Melbourne, Vic Australia

## Abstract

**Purpose:**

The purpose of this randomised controlled trial was to assess the impact of skin incision location on the patients’ ability to kneel.

**Methods:**

A total of 29 patients undergoing bilateral total knee arthroplasty (58 knees) were randomised to receive a lateral or midline incision, with the contralateral limb receiving the alternative option. Cruciate retaining implants were used in all cases by three experienced arthroplasty surgeons. The primary outcome measures assessed functional ability to kneel using an innovative five-point kneeling scale, preferred knee to kneel on and the area of cutaneous sensory loss around the incision at 6 weeks, 6 months and 12 months. Secondary outcome measures were the OKS, KOOS JR, FJS and EQ5D patient reported outcome measures (PROMS), length of surgical scar, overall knee preference and range of motion (ROM).

**Results:**

There were no significant differences between the two groups for any primary or secondary outcome measures. Flexion range however, had a significant positive correlation with kneeling score (*r* = 0.335, *p* = 0.010). The kneeling score increased at each time point after surgery and was significantly greater at 12 months than preoperatively (2.7 v 3.5, *p* = 0.015). The area of sensory loss lateral to the incision was significantly less at 6 and 12 months than at 6 weeks (43.6cm^2^ and 40.1cm^2^ v 84.1cm^2^, *p* < 0.0001).

**Conclusion:**

The ability to kneel following cruciate retaining total knee arthroplasty is not affected by the incision position but by time and flexion range. TKA improves the ability to kneel by 12 months post-surgery. Sensory loss lateral to the incision reduces with time.

**Level of evidence:**

Therapeutic Level 2.

## Introduction

Despite advances in surgical techniques and implants, up to 20% of patients express dissatisfaction following a total knee arthroplasty (TKA) [1], with kneeling ability being rated as the worst functional outcome [2]. This is important as most patients expect being able to perform activities of daily living (ADLs) without limitation following surgery [3]; however, kneeling is not routinely achieved, with between 50–80% of patients reporting difficult or impossible kneeling [4]. Sensory loss (dysaesthesia) over the anterior knee following TKA, occurring as a result of the disruption of the infrapatellar branch of the saphenous nerve, is one reported factor affecting patient kneeling ability [5].

Muller proposed a lateral skin incision to preserve both nerve and blood supply to the lateral flap [6]. Several randomized controlled trials (RCTs) have compared midline or medial to lateral incisions and the area of cutaneous dysesthesia [7–10], all of them concluding that the anterolateral skin incision provided a smaller area of cutaneous alteration [7–10]. However, only one of those RCTs assessed the relationship between cutaneous dysesthesia and kneeling ability, by comparing anterolateral to anteromedial incisions [10], but did not include centred midline incisions, the most frequently employed in TKA surgery [11]. The authors of this study hypothesize that anterior knee skin sensation and thus, kneeling ability, are not influenced by the type of incision.

The objective of this RCT is to compare midline and anterolateral skin incisions in patients undergoing simultaneous bilateral TKA to test the hypothesis that the latter approach would improve kneeling ability and reduce dysesthesia.

## Materials and methods

This was a prospective, two-arm, parallel-group, randomized controlled trial involving patients scheduled for bilateral TKA. Patients were randomly assigned to receive a lateral or midline incision for one of the knees, the other receiving the alternative incision. Three surgeons performed the surgeries following Human Research Ethics Committee approval at the Mater Health Services North QLD Ltd (MHS20161122-01). This study was registered prior to commencement with Australian and New Zealand Clinical Trials Registry (ANZCTRN 12616001706460).

### Participants and selection criteria

Participants scheduled for elective bilateral TKA were invited to participate in this study, and informed consent was obtained upon enrolment. Inclusion criteria included: (1) bilateral knee osteoarthritis suitable for cruciate-retaining total knee arthroplasty and (2) physiological fitness to undergo bilateral simultaneous surgery. Exclusion criteria included: (1) diagnosis other than osteoarthritis, (2) prior trauma or surgical procedures to the knees (3) central or peripheral neurological deficits and (4) knee paraesthesia. All procedures were performed by one of three consultant orthopaedic surgeons (PM, LM, MW) at a single institution. Post-operative follow-up and rehabilitation protocols remained the same for all patients.

### Enrolment, follow-up, and patient characteristics

**Fig. 1:**
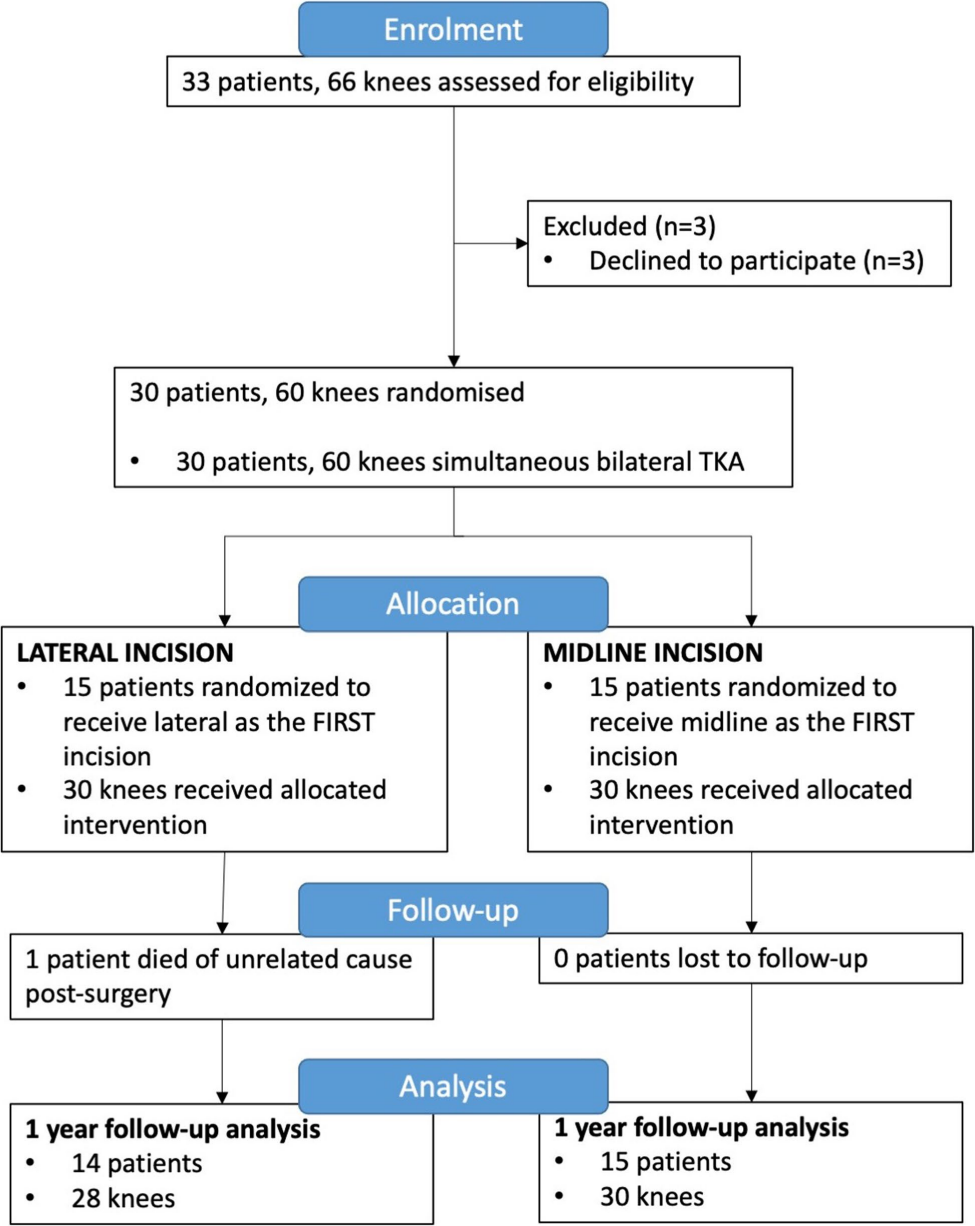
Study flowchart

Patients were enrolled between September 2016 to September 2019. A total of 33 patients (66 knees) were assessed for eligibility and 30 patients (60 knees) were included for randomization. Three patients were excluded; all withdrew for personal reasons prior to surgery. One participant died of an unrelated cause during the first year follow up period (Fig. 1).

### Randomisation and blinding

Randomization was performed at the time of patient consent during the initial surgical consult. The participants identified their most symptomatic knee which was randomised using computer generated random allocation to receive either a midline or lateral incision; the contralateral knee was automatically assigned to the alternative. Blinding of the participants and the clinical outcome assessors was not possible due to the overtly visible surgical incision.

### Surgical procedure

Bilateral simultaneous TKAs were performed as per normal routine by the three surgeons. General or spinal anaesthesia was selected at the discretion of the anaesthetist. Antibiotic prophylaxis cefazolin (2gr.) and tranexamic acid (1gr.) were administered intravenously pre-operatively, and intra-operative periarticular blocks were performed as previously described [12]. A thigh tourniquet was routinely used during the surgical procedures.

Cruciate-retaining, fixed-bearing implants with hybrid fixation (cementless femur, cemented tibia) were used in this study; one surgeon selected *Triathlon* (Stryker Orthopaedics, Mahwah, NJ), using an imageless computer navigation system (Precision CAS eNact Knee Navigation System v4.0 software; Stryker Leibinger, Freiburg, Germany), and the other two surgeons utilised *Persona* (Zimmer Biomet Orthopaedics, Warsaw, IN) using Image Derived Implantation (IDI) technology. Patellae were routinely resurfaced with *Triathlon* implants, and selectively with *Persona*; however, those who had patella resurfacing had this done so bilaterally (i.e., both knees of a patient were treated in the same terms, resurfaced or not).

**Fig. 2:**
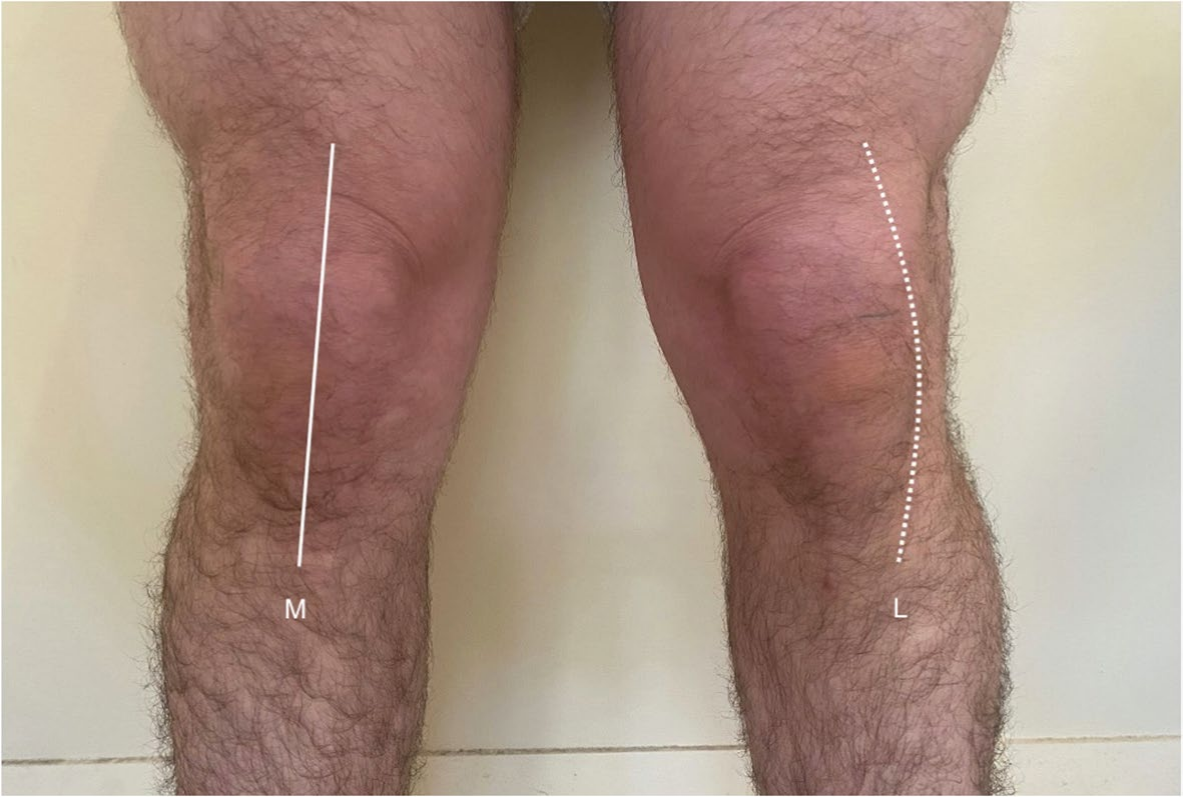
Midline (M) and lateral (L) incisions

Incisions were executed according to the described randomization. The midline incision was centred over the patella extending proximally 5-8cm above the superior border and distally towards the medial aspect of the tibial tuberosity. The anterolateral incision was centred 1cm laterally to the patella, extending 5-8cm above the superior border and distally towards the lateral aspect of the tibial tuberosity as previously described [7] (Fig. 2). The skin incision in both groups was lengthened as required to allow for adequate exposure. Sharp dissection of subcutaneous fat and bursa was performed in both the lateral and midline skin incision groups. The fascia was incised in line with the skin incision, and subfascial dissection was performed to complete appropriate soft tissue flaps and allow exposure for a medial parapatellar arthrotomy, which was performed in all cases. Bony cuts, soft tissue adjustments, trial reduction and definitive component implantation were performed as per surgeons’ routine. A layered closure was performed at the end of the procedure.

### Data collection and outcome measures

**Fig. 3:**
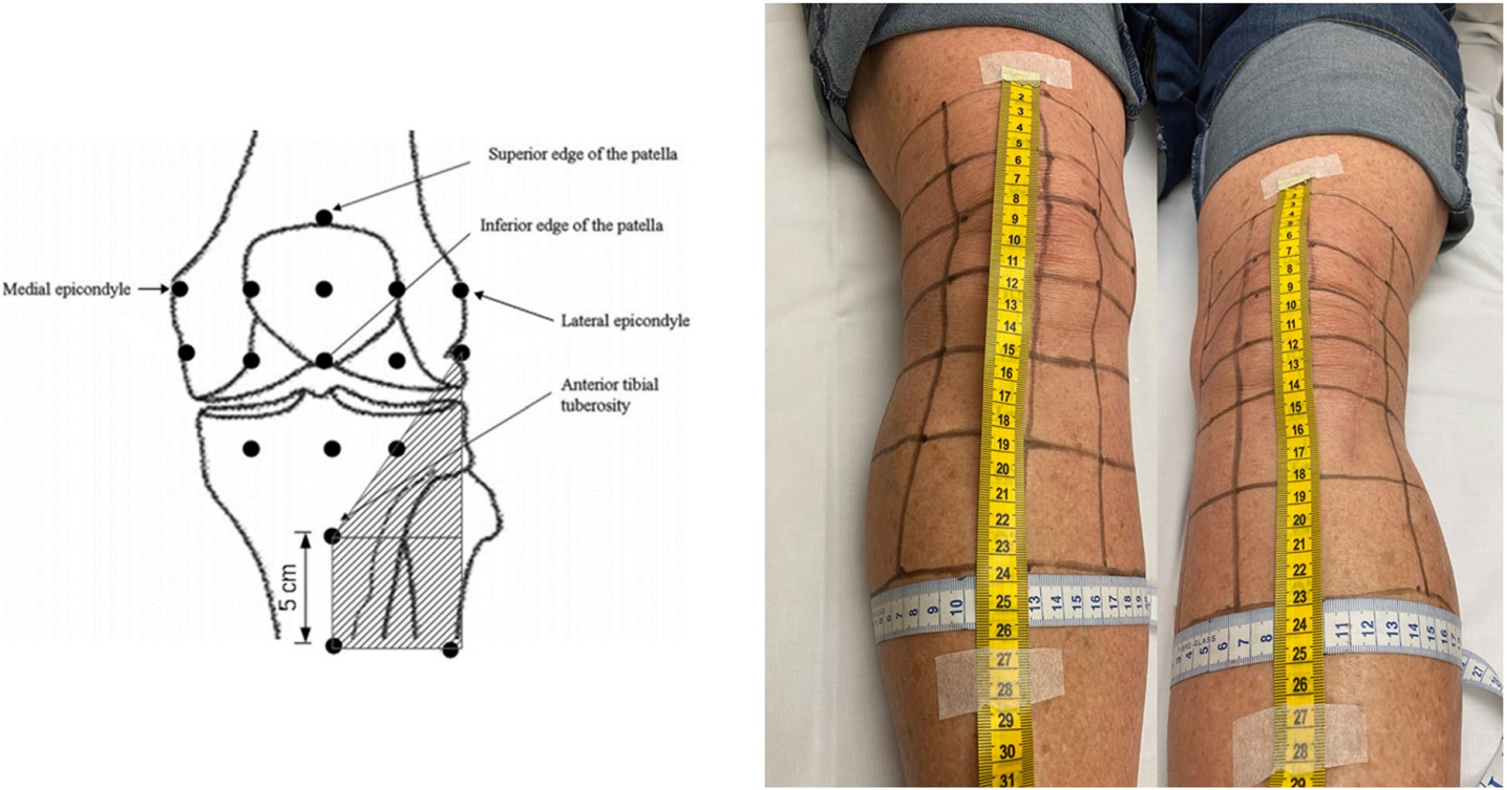
Cutaneous sensory loss assessment

**Fig. 4:**
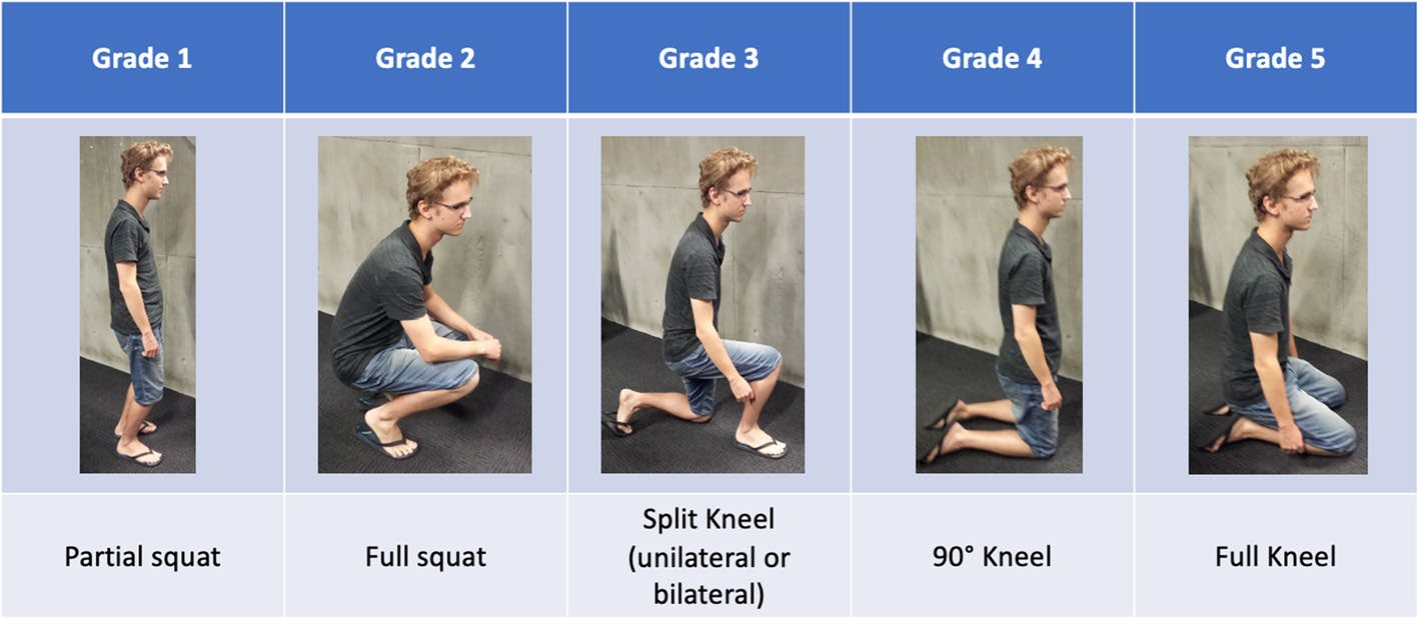
ORIQL kneeling grading system

Patients were followed up at 6 weeks (6w), 6months (6m) and 12 months (12m) post operatively. Primary and secondary outcomes were measured over the reported time points illustrated in Table 1;

- **Primary outcome measure:**
  - *Objective cutaneous sensory scores*: determined using a 5.07/10g Semmes–Weinstein monofilament. A purpose designed grid system to map the anterior knee was utilised and each quadrant was tested for alteration in sensation (Fig. 3). Quadrants identified to have altered sensation were marked and an overall cutaneous surface area of sensory alteration was calculated. The quadrant markings are based on the anatomical landmarks of patients knee and photographed with and without a tape measure to ensure the markings could be replicated between time points per patient. Patients were blinded to cutaneous assessment to reduce the risk of bias.
- **Secondary outcome measures:**
  - *Kneeling ability*: patients were assessed in a progressive fashion from partial squat to a full kneel on both knees utilising an innovative system (Fig. 4). Their best performance was graded according to the following classifications: partial squat = 1, full squat = 2, split kneel with one knee in contact with the ground = 3, 90 degree kneel on both knees = 4, and full kneel with the patient resting on the heels of their feet = 5. A chair was provided to ensure assessment of kneeling ability was not confounded by other co-morbidities impacting this activity.
  - *Clinical evaluation*: this was performed using patient reported outcome measures (PROMs), which included the Knee Injury and Osteoarthritis Outcomes Score (KOOS), Oxford Knee Score (OKS), EuroQol (EQ-5D 3L) and the Forgotten Joint Score (FJS).
  - *Knee preference*: Patients were asked the following question: *Do you have a preferred knee?*” with possible answers “neither” “midline incision knee” or “lateral incision knee”.
  - *Scar length*: measured in full extension, in centimetres.
  - *Range of motion (ROM)*: Passive ROM was assessed using a digital goniometer (GonioMeter Plus, Version 1.0 Alexey Briley). Maximal flexion and extension were recorded, and the resulting value from (max flexion – max extension) was defined as *flexion range*.

**Table 1:** Primary and secondary outcome measures over 12 month post operative period

	**Time points**			
	**Pre-Surgery**	**6 weeks**	**6 months**	**12 months**
**Informed consent & Initial clinical examination**	X			
**Primary outcome:**				
**Sensation assessment**	X*	X	X	X
**Secondary outcomes:**				
**Kneeling assessment**	X	X	X	X
**OKS**	X	X	X	X
**KOOS JR**	X	X	X	X
**FJS**	X	X	X	X
**EQ5D**	X	X	X	X
**Knee preference**	X	X	X	X
**Scar length**		X	X	X
**Range of movement**	X	X	X	X

^*^No assessment however subjects reporting paraesthesia were excluded

### Sample size/power calculation

Assuming Type I error (alpha) to 5% (*p* = 0.05) and Type II error (beta) to 0.2 (power equal to 80%), the sample size calculated was 30 knees in each group for the primary outcome measure based on a previous study [13]. To compensate for expected dropouts, the sample size was increased by 10%, with a total of 66 knees (bilateral TKAs; 33 patients required for each arm).

### Statistical analysis

All data was assessed using the Statistical Sciences (SPSS, Version 22). According to the Shapiro Wilks test, most of the parameters were not normally distributed, and thus the measure of central tendency and dispersion was reported as median and inter- quartile range and non-parametric tests were used for all continuous parameters. Man-Whitney U test was used to compare sensory loss, flexion range and knee flexion between the midline and lateral incisions. A Friedman test was used to determine differences between time points for both midline and lateral incisions. Once main effects of time were identified, a Dunn’s test was conducted as post hoc to ascertain the location of differences. The kneeling scores were classified as ordinal parameters, thus Chi-squared tests were used to compare kneeling scores between the midline and lateral incisions. The correlation between kneeling score and knee flexion was examined using Spearman’s Rho at pre-op, 6w, 6m and 12m. The alpha level was set at 0.05 for all tests.

## Results

**Fig. 5:**
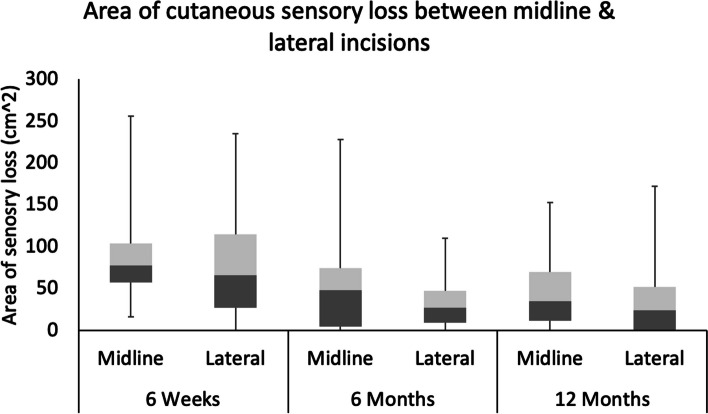
Comparison of primary outcome according to incision type

The cohort of patients and their baseline characteristics are summarized in Table 2. No significant differences between midline and lateral incisions were observed for sensory loss at 6w, 6 m and 12 m (Fig. 5). There was no significant association between incision placement and kneeling ability at pre-op, 6w, 6m and 12m (Table 3). When comparing values between kneeling scores and knee flexion, there was a significant correlation at pre-op and 12m. However, no other correlations were identified at 6w and 6m.

**Table 2:** Median, inter-quartile range and frequency of the baseline characteristics

	**Values**	
**Age (years)**	68 (63–74)	
**Sex (Male/Female)**	F (48%) M (51%)	
**Height (cm)**	1.70 (1.60–1.77)	
**Weight (kg)**	89.0 (82.0–100.0)	
**Body Mass Index, kg/m**^**2**^	31.8 (28.4–35.1)	
	**Midline Incision**	**Lateral Incision**
**Duration of surgery (min)**	65 (57.5–83.5)	65 (59.5–74.5)
**Tourniquet time (min)**	38 (30–45.8)	41.5 (30–46)

**Table 3:** Kneeling scores for the midline and lateral incisions at pre-op, 6 weeks, 6 months and 12 months post-op time points

Time points	Kneel Score	Midline	Lateral	Total	Chi square
**Pre-Op**	Partial squat	11 (50%)	11 (50%)	22	(χ2 = 1.68; *n.s.*)
	Full squat	2 (33.3%)	4 (66.7%)	6	
	Split knee	3 (75%)	1 (25%)	4	
	Kneel 90	9 (50%)	9 (50%)	18	
	Full kneel	4 (50%)	4 (50%)	8	
**6 weeks**	Partial squat	14 (50%)	14 (50%)	28	(χ2 = 1.69; *n.s.*)
	Full squat	8 (61.5%)	5 (38.5%)	13	
	Split knee	3 (33.3%)	6 (66.7%)	9	
	Kneel 90	4 (50%)	4 (50%)	8	
	Full kneel	0 (0%)	0 (0%)	0	
**6 months**	Partial squat	7 (50%)	7 (50%)	14	(χ2 = 0.90; *n.s.*)
	Full squat	6 (60%)	4 (40%)	10	
	Split knee	3 (37.5%)	6 (62.5%)	8	
	Kneel 90	11 (50%)	11 (50%)	22	
	Full knee	2 (50%)	2 (50%)	4	
**12 months**	Partial squat	2 (50%)	2 (50%)	4	(χ2 = 0.42; *n.s.*)
	Full squat	6 (54.5%)	5 (45.5%)	11	
	Split knee	1 (33.3%)	2 (66.7%)	3	
	Kneel 90	16 (50%)	16 (50%)	32	
	Full knee	4 (50%)	4 (50%)	8	

**Fig. 6:**
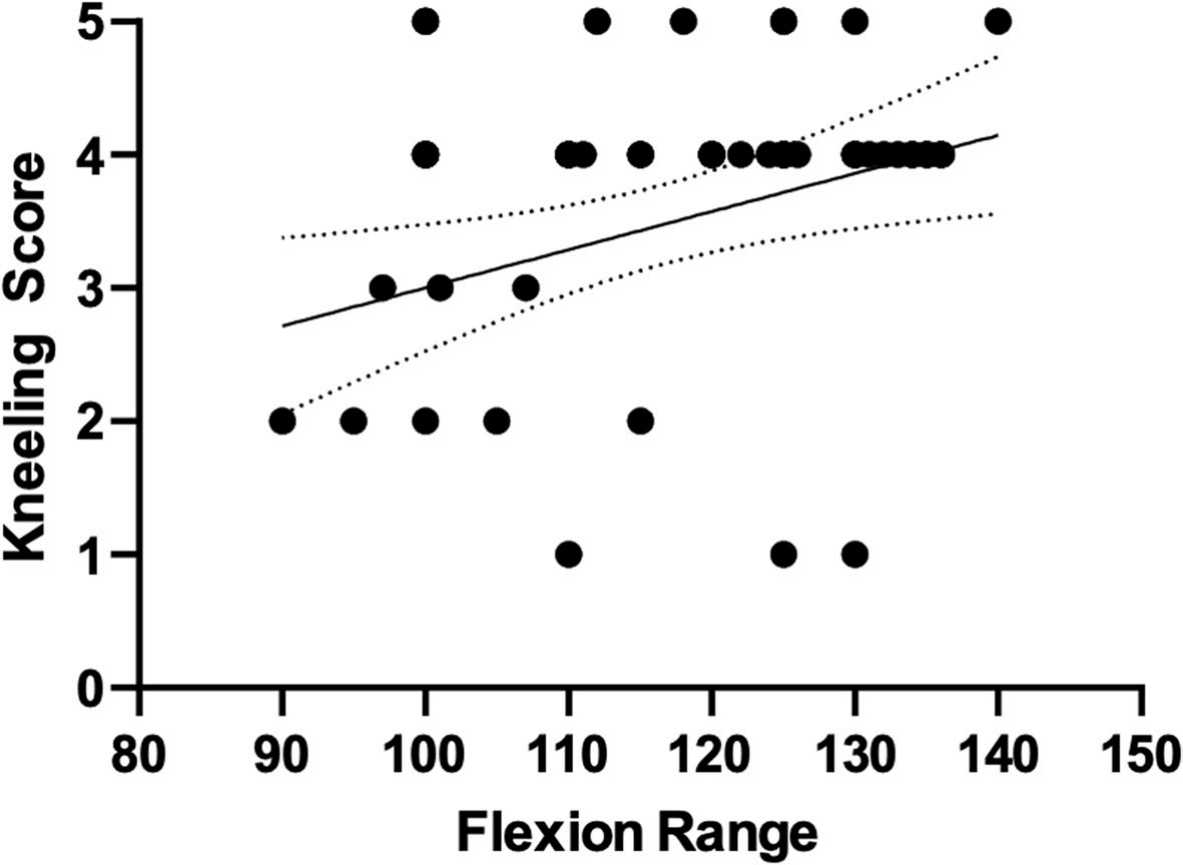
Diagram showing correlation between kneeling scores and flexion range

Knee flexion had a significant positive correlation with kneeling score (Fig. 6). The kneeling score increased at each time point after surgery and was significantly greater at 12 m than preoperatively.

PROMs improved in each individual incision group from pre-operative to 12 m, with no statistically significant difference between incision groups. No difference was either determined for incision length, knee preference or ROM (Table 4).

**Table 4:** Secondary outcome measures expressed as median and IQR

	Time Point	Anterolateral Incision	Midline Incision	*P *value
**KOOS**	Pre-operative	47 (13)	50 (15)	*n.s*
	1 Year	92 (20)	92 (24)	*n.s*
**EQ5D**	Pre-operative	70 (20)	70 (20)	*n.s*
	1 year	90 (15)	90 (18)	*n.s*
**OKS**	Pre-operative	18 (17)	17 (16)	*n.s*
	1 year	45 (20)	45 (20)	*n.s*
**FJS**	6 Weeks	20 (34)	19 (30)	*n.s*
	6 Months	58 (29)	63 (50)	*n.s*
	12 Months	77 (31)	79 (21)	*n.s*
**Length of scar (cm)**	Post-operative	16 (15–17)	17 (15–18)	*n.s*
**Patient knee preference (%)**	Post-operative	24%	17%	*n.s*
**Range of movement (degrees)**	Pre-operative	110 (95–125)	115 (94–129)	*n.s*
	12 months	120 (101–127)	114 (101–125)	*n.s*
**Maximal flexion (degrees)**	Pre-operative	116 (105–125)	121 (105–130)	*n.s*
	12 Months	116 (102–130)	116 (100–125)	*n.s*

**Fig. 7:**
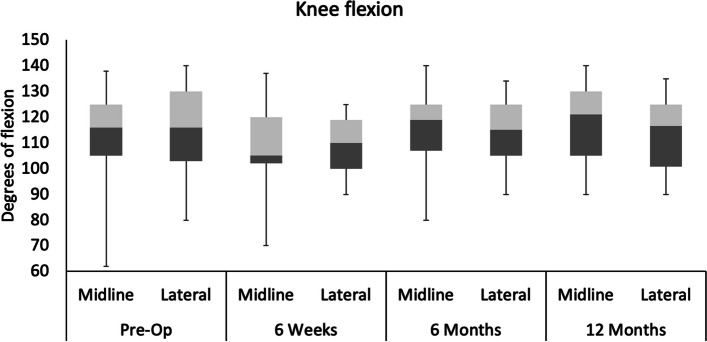
Knee flexion during study time points

**Fig. 8:**
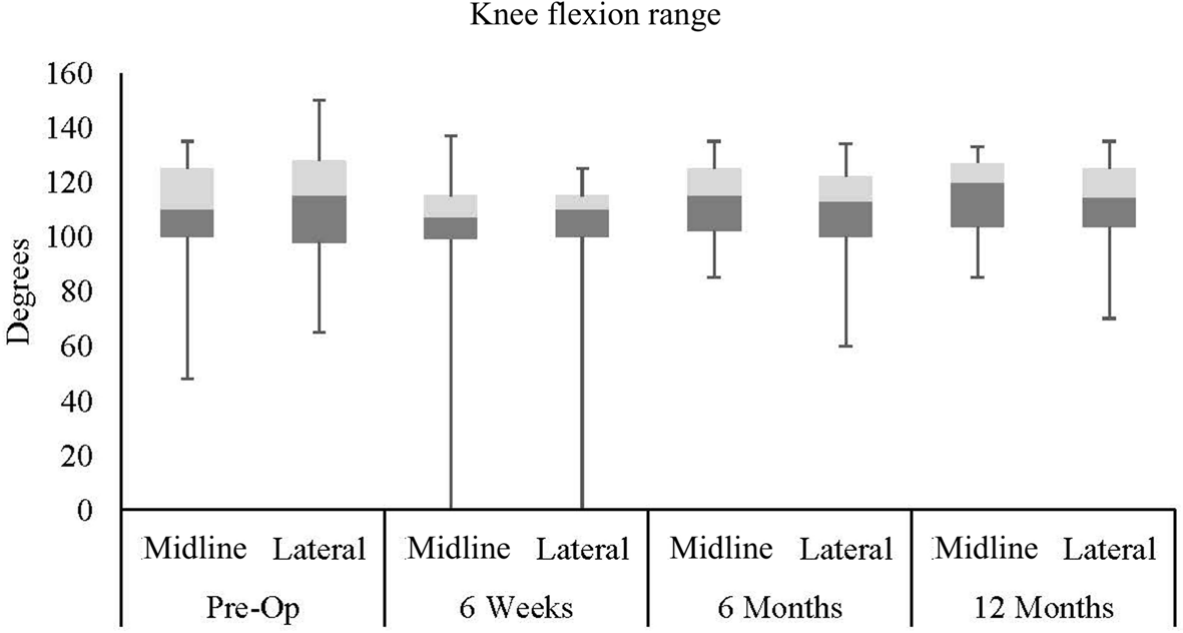
Knee flexion range during study time points

No differences were found between incisions for knee flexion range and knee flexion at pre-op, 6w, 6 m and 12 m (Figs. 7 and 8). However, significant main effects of time were identified for sensory loss, knee flexion range and knee flexion. Posthoc analyses revealed that sensory loss was significantly lower at 6 m and 12 m when compared to 6w for midline and lateral incisions. When comparing knee flexion range at 6w, values were significantly greater at 6 m and 12 m for midline incision, whilst values were only significantly greater at 12 m for lateral incision, with no differences between 6w and 6 m. For knee flexion, values for midline incision were significantly greater at 6 m and 12 m when compared to 6w. However, knee flexion was significantly greater only at 12 m compared to 6w for lateral incision, with no differences between 6w and 6 m.

## Discussion

The most important finding of this study was that the ability to kneel following cruciate retaining TKA was not affected by the position of the incision but by time and flexion range. TKA improves the ability to kneel at 12 m and cutaneous sensory disturbance reduced with time regardless of incision type. The results also demonstrated that cosmetically, there is no statistically significant difference in the overall length of scar when comparing the two groups. To our knowledge, this is the first simultaneous bilateral TKA RCT comparing midline to anterolateral incisions in terms of sensory loss and kneeling ability.

These findings contrast with previous evidence suggesting that an anterolateral skin incision provided a smaller area of cutaneous dysesthesia and higher rates of kneeling ability [8, 10, 14] [11]. However, another study concluded that after three years of mean follow-up, residual numbness following TKA surgery is not a predisposing factor for low kneeling ability or subjective dissatisfaction [15].

A plausible explanation for the contrasting results is that, in the current study, patients were blinded when cutaneous sensation was being assessed, which was not followed in the previous works [7, 8, 10]. In addition, a wide assessment methodology has been employed among other studies; in the current study a purpose designed grid system was used to map the anterior knee anatomy [7], while others have used computer image software, which may be more sensitive in detecting smaller changes. Nevertheless, accurately determining sensory alterations after TKA has been appointed as a structural flaw in this field of research [15], and could be behind the contradictory findings in relation to dysesthesia affecting [5, 16] or not [17] kneeling ability.

The present results demonstrated that both groups reported reduced cutaneous dysesthesia at 12 m, which is in keeping with previous reports [7, 18–20]. Thus, sensory disturbance of the anterior knee skin is expected to improve over time. It has been suggested that in case of persistent tenderness after TKA, the presence of a subcutaneous neuroma should be ruled out, and surgically excised if found [5].

A less known alternative for TKA surgical approach is the transverse incision. Ojima et al. communicated lower sensory disturbance, improved cosmesis and better kneeling performance after TKA performed under transverse incision, compared to a longitudinal midline approach, with similar surgical times and wound complication rates [21]. A 2021 meta-analysis concluded that patients with a transverse incision had increased kneeling odds compared to those with a longitudinal incision [22].

Kneeling ability after TKA is multifactorial, and to a certain extent, controversial [22–25]. Wilding et al. observed that the combination of a CR design and a resurfaced patella showed the better kneeling outcomes, compared to all other combinations involving PS/CR and resurfaced/unresurfaced patellae [25]. This contrasts with other results reporting better midterm kneeling odds with unresurfaced patellae [26], and a recent review found no association between kneeling ability and patellar resurfacing or knee flexion range [4]. A flexion goal of 120º has been set to maximise kneeling ability, too [23]; a recent RCT concluded that, although no implant design was able to restore normal knee kinematics, CR-rotating platforms and PS designs allowed for better knee flexion [27]. Scott et al. reported extension and increased sagittal offset of the femoral component as factors associated with poorer kneeling odds [28]. A large study concluded that men and patients with low BMI or with occupations/hobbies requiring kneeling are more likely to kneel [29]. Smith et al. observed that, although knee pain fairly associated with inability to kneel, in a majority if patients it was not neuropathic but nociceptive [24].

Nevertheless, some evidence also suggest kneeling may not represent an issue of primary concern for surgeons and patients. Series reporting low kneeling ability generally consider it as a yes/no dichotomy, neglecting the range of positions disclosed in Fig. 4, and therefore underestimating functional kneeling [28]. This is also in keeping with the observation that perceived inability to kneel significantly deviated from objectively assessed kneeling [23, 25, 30]; Amin et al. argued that this misperception may be "*the greatest limitation to kneeling after TKA implantation*" [30]. Despite difficulty kneeling tends to appear soon after the operation and remain relatively unchanged with the passing of the years [2], kneeling improvement can be achieved with training [5]; 81% of patients who were unable to kneel at 18–24 months postoperatively, attained functional kneeling after a 6-week desensitization home-based protocol [31]. Furthermore, a clarifying study concluded that in the long term, patients tend to accept poor kneeling capability as their knee function is overly improved by TKA, and often find adaptations for their activities [2]. Perhaps, a more comprehensive assessment of kneeling, considering objective functional evaluation [32], functional/emotional/social impact on patients and expectations may provide a deeper understanding of this subject [4].

This study has several limitations. Our open-label study design meant that it was not possible to blind the surgical team, or the assessors to cutaneous sensation and kneeling ability. Furthermore, pre-operatively cutaneous sensation was not assessed, and therefore baseline comparation was not feasible. However, gross knee paraesthesia was considered an exclusion criterion, so the potential effect of this omission has been considered as low. Another limitation has been sample size; the proposed size (*n* = 30) was not reached by only one patient; nonetheless, significant improvements in flexion range, kneeling ability and dysesthesia were observed. Another potential shortcoming of this study is the limited 12 m follow-up, as numbness can revert during the first two years [16] and kneeling ability has been observed to improve over time at least until the 36 m mark [22]. However, surveillance and follow-up compliance after the first year can be as low as 35% [33]. Lastly, the coexistence of different implants and variable patellar resurfacing may be a potential cofounding factor; bilaterality may mitigate this consideration, though.

The results of this study have several clinical implications. Surgeons can be reassured that regardless surgical incision preference, kneeling ability and dysesthesia improve with time in a comparable manner. When discussing TKA with patients, the results from the present study can be utilised as part of expectation management, which is commonly the major contributing factors toward TKA dissatisfaction [1]. Patients should be encouraged to actively achieve high knee flexion, as this has been identified as directly related with better kneeling ability.

## Conclusions

This study suggests that the ability to kneel following cruciate retaining bilateral TKA is not affected by the position of the incision, but rather by time and flexion range. To ensure the patient can kneel after a knee replacement, it is important to maximise flexion range postoperatively. Sensory loss lateral to the incision appears to reduce with time regardless of incision type. Kneeling ability is complex and dependent on multiple factors, not just the surgical incision and/or anterior knee dysesthesia.
